# Combined Supplementation of Live Yeast and Yeast Postbiotics Enhances Antioxidant Capacity and Intestinal Health in Weaned Piglets

**DOI:** 10.3390/antiox15050623

**Published:** 2026-05-14

**Authors:** Xiangshi Luo, Pingping Xu, Shukai Cao, Zhengcheng Zeng, Shupeng Wang, Hao Zhang, Tadele Kiros, Shuai Zhang

**Affiliations:** 1 State Key Laboratory of Animal Nutrition, College of Animal Science and Technology, China Agricultural University, Beijing 100193, China; luoxiangshi@cau.edu.cn (X.L.); sy20253041085@cau.edu.cn (S.C.); zengzhengcheng@cau.edu.cn (Z.Z.); s20253040903@cau.edu.cn (S.W.); 2 College of Veterinary Medicine, Nanjing Agricultural University, Nanjing 210095, China; xupingping@stu.njau.edu.cn (P.X.); zhanghao@stu.njau.edu.cn (H.Z.); 3 Phileo by Lesaffre North America, 7475 West, Main Street, Milwaukee, WI 53214, USA; t.kiros@phileo.lesaffre.com

**Keywords:** antioxidant capacity, intestinal morphology, live yeast, yeast postbiotics, weaned piglets

## Abstract

Weanling piglets, with immature immune system and physiological functions, often experience post-weaning diarrhea. This study investigated the effects of combined supplementation of live yeast and yeast postbiotics on the growth performance, antioxidant capacity and intestinal health of weaned piglets. A total of 224 weaned piglets (22 days old) were randomly assigned to four groups: a basal diet (CON) or the same diet supplemented with 1600 ppm zinc oxide (ZnO), high-dose live yeast and yeast postbiotics (LYYP-H), or low-dose live yeast and yeast postbiotics (LYYP-L). The trial lasted 28 days, with diets divided into 2 phases. The results showed that dietary combined supplementation with live yeast and yeast postbiotics did not significantly affect the average daily gain (ADG), average daily feed intake (ADFI), or the diarrhea rate (*p* > 0.05). However, dietary combined supplementation with live yeast and yeast postbiotics significantly enhanced fecal consistency in piglets (*p* < 0.05). Moreover, dietary combined supplementation with live yeast and yeast postbiotics significantly improved representative antioxidant indices, notably superoxide dismutase (SOD) and glutathione peroxidase (GSH-Px), and strengthened immune capabilities; additionally, a marked improvement in intestinal morphology was observed (*p* < 0.05). In conclusion, the combination of live yeast and yeast postbiotics can improve antioxidant capacity and intestinal health and show the potential to replace high doses of ZnO during the first two weeks post-weaning.

## 1. Introduction

Weaning is a critical phase in pig’s life. Due to their immature digestive and immune systems, piglets are highly vulnerable to weaning stress induced by physiological, environmental, and social factors [1]. This stress typically manifests as post-weaning diarrhea, impaired intestinal integrity, and dysbiosis of the gut microbiota [2], which collectively diminish long-term growth performance and increase mortality rates. Particularly under intensive production condition, weaning occurs much earlier than in natural environments. Such precocious weaning compromises intestinal barrier function, with empirical evidence linking the severity of barrier impairment to the earliness of weaning [3]. Consequently, nutritional interventions through functional feed additives have emerged as a primary strategy for mitigating these adverse effects [4]. Historically, antibiotics and pharmacological level of zinc oxide (ZnO) were extensively utilized due to their efficacy in controlling diarrhea. However, antibiotic misuse has precipitated antimicrobial resistance, posing a severe threat to public health [5]. In response, China has implemented a comprehensive ban on antibiotics as growth promoters in animal feed since 2020. Furthermore, prolonged exposure to high-dose ZnO induces toxic accumulation in the hepatic and renal tissues, while the excretion of zinc-rich feces entails significant environmental externalities [6]. Since these substances were traditionally the primary pharmacological tools used to mitigate post-weaning gastrointestinal disorders, their restriction leaves piglets highly vulnerable to the multifaceted challenges of weaning stress. Therefore, developing safe and efficient alternatives to alleviate weaning stress is now a paramount objective in livestock industry. Recent scholarly attention has increasingly shifted toward novel additives such as probiotics and postbiotics [7], with a specific emphasis on live yeast and yeast postbiotics. While several studies have documented the beneficial effects of either live yeast or yeast postbiotics on livestock performance [8,9], comparative analyses regarding their efficacy as alternatives to pharmacological levels of ZnO remain limited. Furthermore, the specific mechanisms by which the combination of live yeast and yeast postbiotics mitigates weaning stress are not yet fully elucidated.

Live yeast, a unicellular facultative anaerobic fungus, is a potent probiotic that modulates immune response, modifies gut microbiota, enhances systemic antioxidant capacity, and optimizes intestinal health [10]. Within these taxa, *Saccharomyces cerevisiae* is extensively documented across diverse livestock species [10]. In addition, yeast postbiotics consist of inanimate yeast cells and their metabolites. These postbiotics contain various bioactive compounds, including cell wall polysaccharides like β-glucans and mannan-oligosaccharides (MOS). These polysaccharides bind to pathogens to competitively inhibit their attachment and colonization within the gastrointestinal tract. Extant literature suggests that these compounds improve fecal consistency and reduce diarrhea incidence in weaned piglets, while also modulating gut microbiota and down-regulating the expression of inflammation-related genes, thereby enhancing overall growth performance [11]. Furthermore, metabolic constituents, particularly short-chain fatty acids, are pivotal in augmenting microbial diversity and modulating gut microbiota [12]. Beyond physiological efficacy, yeast postbiotics offer distinct logistical advantages over live yeast, exhibiting superior stability during industrial processing, transportation, and protracted storage [13].

We hypothesize that this dietary combined supplementation with live yeast and yeast postbiotics will alleviate post-weaning diarrhea, enhance antioxidant capacity, and improve intestinal health, thereby enhancing growth performance in weaned piglets. Therefore, the present study was conducted to evaluate the efficacy of combined live yeast and yeast postbiotics as functional alternatives to pharmacological doses of ZnO by systematically assessing their impacts on growth performance, diarrhea incidence, antioxidant capacity, and intestinal health in weaned piglets.

## 2. Materials and Methods

### 2.1. Animals and Dietary Treatments

A total of 224 healthy weaned pigs [Duroc × (Landrace × Yorkshire), 22 days old] with average initial body weight of 6.62 ± 1.19 kg were randomly assigned to four dietary treatments. There were 8 pigs (4 barrows and 4 gilts) per pen and 7 replicate pens per treatment. The experiment was conducted at the animal experimental base of China Agricultural University, located in Chengde, Hebei Province, China. The weaned piglets were housed in 1.2 × 2.1 m^2^ pens equipped with plastic slatted floors, automatic stainless steel nipple drinkers, and feeders. Initial room temperature was maintained at 28 °C for the first two weeks and then gradually reduced by 1 °C weekly, and the relative humidity was controlled at 60 % to 70 %. The pens and barn were cleaned by broom every day to keep the environment sanitary as well as prevent some diseases from spreading among the pigs. All piglets had free access to feed and water ad libitum throughout the 28-day trial period.

The experiment was divided into two phase diets and piglets were fed either basal diet I only (CON) or basal diet I supplemented with either 1600 ppm ZnO (ZnO), 750 g/t live yeast + 500 g/t yeast postbiotic (LYYP-H), or 500 g/t live yeast + 300 g/t yeast postbiotic (LYYP-L) from d1-d14. In Phase II (d15-28), CON and ZnO groups received basal diet II only, while the other two groups received basal diet II supplemented with lower doses of the probiotic and postbiotic products (500 g/t live yeast + 300 g/t yeast postbiotic (LYYP-H), and 350 g/t live yeast + 300 g/t yeast postbiotic (LYYP-L). The experimental design followed a two-phase feeding program to simulate commercial production cycles. High levels of supplementation were applied in Phase I to address the peak period of weaning-induced challenges. The transition to Phase II reflected both regulatory and economic considerations. For the ZnO group, extra supplementation was discontinued in Phase II to adhere to Chinese national regulations (MARA Announcement No. 2625), which limit total Zn to 110 mg/kg after the initial 14-day post-weaning period. Considering that the basal diet II across all groups already contained 105.78 mg/kg of Zn, no supplemental ZnO was added to the ZnO group during Phase II. For the LYYP groups, dosage was adjusted in Phase II to evaluate a cost-effective application strategy consistent with industry standards as piglets transition toward physiological maturation. The yeast probiotics *Saccharomyces cerevisiae* CNCM I-4407 (Actisaf Sc 47 HR+^®^) and postbiotics yeast cell wall fraction rich in mannan-oligosaccharides (Safmannan^®^) are proprietary products of Phileo by Lesaffre (Marcq-en-Baroel, Lille, France). The diets were formulated to meet or exceed the nutrient requirements of pigs outlined in NRC (2012), and the ingredients composition of two basal diets are presented in Table 1.

### 2.2. Sample Collection

Body weight of individual piglet was measured at the beginning (0 d), middle (15 d), and end (29 d) of the trial after an overnight fast. The feed consumption by pen was recorded every day. Average daily gain (ADG), average daily feed intake (ADFI), and the ratio of feed intake to gain (F:G) were calculated based on these dates.

Diarrhea scores were assessed visually and recorded every day (0 = normal; 1 = pasty; 2 = semi-liquid; 3 = watery). We considered diarrheic when scored for 2 and 3, and considered unformed when scored for 1, 2 and 3. The diarrhea incidence (%) were computed using the following equations:

Diarrhea rate (%) = (the diarrhea days × the number of diarrhea pigs)/(the total experiment days × the total number of pigs) × 100

Unformed feces rate (%) = (the unformed days × the number of pigs with unformed feces)/(the total experiment days × the total number of pigs) × 100

On the morning of day 15 and day 29, 28 pigs (one pig per replicate, whose body weights were closest to the pen average) were selected. Blood samples were collected in 10 mL vacutainer tubes via anterior vena cava. Blood was centrifugated at 3000× *g* for 15 min at 4 °C. Serum samples were stored at −20 °C and analyzed within one month after collection. Then the selected piglets were euthanized by intraperitoneal injection of sodium pentobarbital. From each of the selected piglets, a 2 cm section of jejunum was taken and promptly fixed in 10% neutral buffered formalin. Then, the remaining jejunal segment was rinsed with normal saline. Two sections were then collected: one was immediately snap-frozen in liquid nitrogen as a full-thickness tissue sample, while the other was opened longitudinally to carefully scrape off the mucosal layer using a glass slide and also snap-frozen in liquid nitrogen. These samples were stored at −80 °C for detection of immune indices and intestinal integrity. Cecum contents were collected in cryopreserved tubes and immediately frozen in liquid nitrogen and stored at −80 °C until testing for microbial composition analysis.

### 2.3. Serum Analysis

The activities of superoxide dismutase (SOD), catalase (CAT), and glutathione peroxidase (GSH-Px), as well as the concentration of malondialdehyde (MDA) in serum, were determined using commercial assay kits according to the manufacturer’s instructions (Nanjing Jiancheng Bioengineering Institute, Nanjing, China). All analyses were performed in technical duplicates.

### 2.4. Intestinal Morphology Analysis

After dewaxing and rehydration, sections of 5 μm thickness were stained with hematoxylin–eosin to assess the morphology of the intestinal wall. In each section, villus height, associated crypt depth were measured using Image-pro plus 6.0 (Media Cybernetics Inc., Rockville, MD, USA). Villus height (VH) was defined as the vertical distance from the villus tip to the villus-crypt junction. Crypt depth (CD) was defined as the vertical distance from the villus-crypt junction to the base of the crypt. At least 10 well-oriented intact crypt-villi were examined in each piglets’ jejunum. The mean villus height and crypt depth in the jejunum were then calculated per piglet.

### 2.5. Intestinal Integrity Analysis

Intestinal mucosa was homogenized in ice-cold phosphate-buffered saline containing a protease inhibitor cocktail (Beyotime Biotechnology, Shanghai, China) and then centrifuged at 10,000× *g* for 10 min at 4 °C. The resulting supernatant was immediately partitioned into multiple aliquots and stored at −80 °C. Each aliquot was thawed only once for the specific measurement of either [diamine oxidase (DAO) and D-lactic acid (D-LA)] or intestinal immune indices to ensure consistent sample handling. DAO activity and D-LA were detected using enzyme-linked immunosorbent assay (ELISA) kits according to the manufacturer’s instructions (Jiangsu Yutong Biotechnology Co., Ltd., Nanjing, China). A Quantitative Real-time PCR kit was used to determine the expression of intestinal tissue tight junction-related genes zonula occludens-1 (*ZO-1*), *Occludin*, and *Claudin-1*. Total RNA was extracted from jejunal sample using the TRIZOL reagent (Invitrogen, Carlsbad, CA, USA). RNA integrity was verified by agarose gel electrophoresis. cDNA was synthesized with PrimeScript RT kit (TaKaRa Biotechnology (Dalian) Co., Ltd., Dalian, China). Real-time PCR was performed using TB Green^®^ Premix Ex Taq^™^ II reagents (TaKaRa Biotechnology (Dalian) Co., Ltd., Dalian, China) and Roche LightCycler^®^ 480II RT qPCR Detection System (F. Hoffmann-La Roche, Ltd., Basel, Switzerland). The target gene primer sequences are shown in Table 2. The specificity of the qPCR products was confirmed by melting curve analysis, which consistently showed a single peak for each gene. To ensure the reliability of the relative quantification, representative amplification and melting curves for all investigated primers on day 14 and day 28 have been provided in the Supplementary Materials (Figures S1 and S2). The consistent slopes and parallelism observed in the amplification curves across all samples indicate a stable and optimized amplification process, supporting the validity of the 2^−ΔΔCt^ method. Finally, the mRNA expression of target gene relative to housekeeping gene (*β-actin*) was calculated by the method of 2^−ΔΔCt^.

### 2.6. Intestinal Immunity Analysis

Following the same protocol, the supernatant was obtained from the jejunal mucosa and aliquoted for subsequent analysis. Then, the concentration of secretory immunoglobulin A (sIgA) and the concentrations of cytokines including interleukin-6 (IL-6), interleukin-10 (IL-10), tumor necrosis factor-α (TNF-α), transforming growth factor-α (TGF-α), transforming growth factor-β (TGF-β) and interferon-γ (IFN-γ) were determined using the corresponding commercially ELISA kits and Standard specification microplate reader (Beijing Laibo Tairui Technology Development Co., Ltd., Beijing, China).

### 2.7. Cecal Microbiological Analysis

16S rRNA sequencing was used to determine the microbial composition. The total DNA was extracted from the samples using the QIAamp Fast DNA Stool Mini Kit (Qiagen, Hilden, Germany) according to the manufacturer’s instructions. The PCR product was extracted from 2% agarose gel and purified using the AxyPrep DNA Gel Extraction Kit (Axygen Biosciences, Union City, CA, USA) according to the manufacturer’s instructions and quantified using a Qubit fluorometer (Invitrogen, Carlsbad, CA, USA). Purified amplicons were pooled in equimolar amounts and paired-end sequenced on an Illumina MiSeq PE300 platform (Illumina, San Diego, CA, USA). Microbial community composition was analyzed via high-throughput sequencing on the Illumina MiSeq platform. Specifically, the hypervariable V3–V4 regions were targeted for PCR amplification using the primers 338F (5′-ACTCCTACGGGAGGCAGCAG-3′) and 806R (5′-GGACTACHVGGGTWTCTAAT-3′). Average sequencing depth was 50,000 reads per sample. The resultant sequences were categorized into operational taxonomic units (OTUs) based on a 97% similarity threshold. Subsequent library construction, sequencing, and bioinformatic analyses were conducted using the NovoMagic Cloud Platform (Novogene, Beijing, China).

### 2.8. Statistical Analysis

Data of growth performance, antioxidant capacity, intestinal morphology, intestinal integrity and intestinal immunity were initially verified for normality and outliers using the UNIVARIATE procedure of SAS 9.4 (SAS Institute Inc., Cary, NC, USA). Subsequently, these data were analyzed by one-way ANOVA using the GLM procedure of SAS, and Tukey’s test was used to adjust the multiple comparisons. Diarrhea incidence (diarrhea rate and unformed feces rate) was analyzed via nominal logistic regression using the LOGISTIC procedure of SAS. For gut microbiota analysis, alpha-diversity indices were calculated at 97% identity and analyzed via the non-parametric Kruskal–Wallis test in SAS. Beta-diversity was investigated with QIIME using principal coordinate analysis (PCoA) based on the weighted UniFrac distance matrix. To determine significant differences in the microbial community, permutational multivariate analysis of variance (PERMANOVA) was performed with the adonis function. For the comparison of microbial taxonomic abundance between two specific groups, the Wilcoxon rank-sum test was employed. To account for the potential for false positives in multiple taxa comparisons at the genus level, *p*-values were subsequently adjusted using the Benjamini–Hochberg False Discovery Rate (FDR) method, with statistical significance defined as an adjusted *p*-value < 0.05.

Each pen served as the experimental unit for growth performance and diarrhea indicators. For all other parameters, the individual pig was considered the experimental unit, as one pig per pen was randomly selected for sampling. *p* ≤ 0.05 was considered statistically significant, and 0.05 < *p* < 0.10 indicated a trend.

## 3. Results

### 3.1. Effects of Combined Supplementation of Live Yeast and Yeast Postbiotics on Growth Performance and Diarrhea Incidence

Results for growth performance and diarrhea incidence are summarized in Table 3. No significant differences were observed in ADG or ADFI among the groups during either phase or over the entire trial period (*p* > 0.05); however, a trend was noted where ADG in the CON group was higher than that of the other groups during Phase II. Similarly, while no significant differences in F:G were found among treatments during Phase I (*p* > 0.05), the LYYP-H group tended to have a higher F:G than the other groups during Phase II. Furthermore, throughout the entire trial period, the F:G in the LYYP-H group was significantly higher than that of the CON and LYYP-L groups (*p* = 0.005). Regarding diarrhea, no significant differences in diarrhea rate were detected among treatments during any stage or the overall period (*p* > 0.05). Notably, the unformed feces rate in the LYYP-H and LYYP-L groups was significantly lower than that of the CON group during Phase II and across the entire trial period (*p* < 0.05), with a similar downward trend observed during Phase I.

### 3.2. Effects of Combined Supplementation of Live Yeast and Yeast Postbiotics on Serum Antioxidant Indices

Serum antioxidant indices are summarized in Table 4. Compared with the CON group, SOD activity was significantly higher in the ZnO and LYYP-H groups on both day 14 and day 28 (*p* < 0.05), while the MDA concentration in the CON group was significantly higher than that of all other groups on day 14 (*p* < 0.05), and remained significantly higher than the LYYP-H group on day 28 (*p* < 0.05). Regarding GSH-Px, activity was significantly elevated in the LYYP-H group compared with the CON and ZnO groups on day 14 (*p* < 0.05); by day 28, GSH-Px activity in the CON group was significantly lower than in all other groups (*p* < 0.05). Additionally, CAT activity in the CON group tended to be lower than in the other groups on both day 14 and day 28.

### 3.3. Effects of Combined Supplementation of Live Yeast and Yeast Postbiotics on Intestinal Morphology

As shown in Table 5, jejunal VH was significantly higher in the ZnO, LYYP-H, and LYYP-L groups than in the CON group on day 14 (*p* < 0.05); by day 28, significant increases remained evident in the ZnO and LYYP-L groups compared with the CON group (*p* < 0.05). In contrast, no significant differences in CD were observed among the groups on either day 14 or day 28 (*p* > 0.05). Consequently, the villus height to crypt depth (V/C) ratio was significantly higher in the LYYP-H and LYYP-L groups relative to the CON group on day 14 (*p* < 0.05), while on day 28, the ZnO and LYYP-L groups exhibited a significantly greater V/C ratio than the CON group (*p* < 0.05).

### 3.4. Effects of Combined Supplementation of Live Yeast and Yeast Postbiotics on Intestinal Integrity

**Figure 1:**
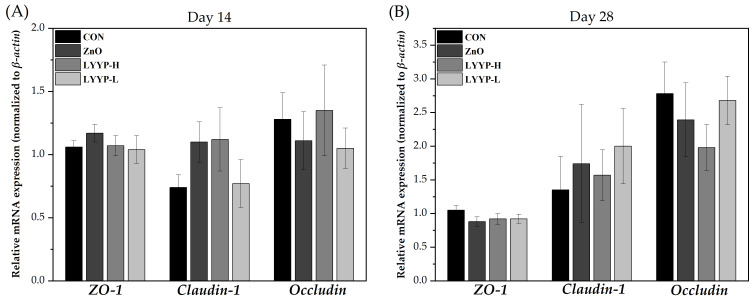
Effects of combined supplementation of live yeast and yeast postbiotics on the relative mRNA expressions of critical genes involved in intestinal tight-junction proteins at (**A**) day 14 and (**B**) day 28. CON, basal diet; ZnO, basal diet supplemented with 1600 ppm ZnO; LYYP-H, basal diet supplemented with high-dose of live yeast and yeast postbiotics; LYYP-L, basal diet supplemented with low-dose of live yeast and yeast postbiotics; *ZO-1*, zonula occludens-1. Error bars represent the SEM. No significant differences or trends were observed between groups for any of the genes on both day 14 and day 28 (*p* > 0.10).

As shown in Figure 1, the relative expression levels of the *ZO-1*, *Claudin-1*, and *Occludin* genes showed no significant differences (*p* > 0.05) between groups on both day 14 and day 28. Similarly, as shown in Table 6, there were no significant differences (*p* > 0.05) in the DAO and D-LA levels on both day 14 and day 28.

### 3.5. Effects of Combined Supplementation of Live Yeast and Yeast Postbiotics on Intestinal Immunity

Intestinal immune indices are summarized in Table 7. Regarding sIgA levels, no significant differences were observed among the groups on day 14 (*p* > 0.05); however, by day 28, sIgA concentrations were significantly higher in the ZnO, LYYP-H, and LYYP-L groups than in the CON group (*p* = 0.003). For IFN-γ, levels in the CON group were significantly higher than those in the ZnO group on day 14 (*p* = 0.017), and remained significantly higher than in the LYYP-H and LYYP-L groups on day 28 (*p* = 0.004). Similarly, TNF-α levels in the CON group were significantly elevated compared with the ZnO and LYYP-H groups on day 14 (*p* = 0.004), and exceeded those of the LYYP-H and LYYP-L groups on day 28 (*p* = 0.002). Additionally, IL-6 levels were significantly higher in the CON group than in the ZnO group on day 14 (*p* = 0.012), and higher than in the ZnO and LYYP-H groups on day 28 (*p* = 0.021). Regarding IL-10, the CON group exhibited significantly higher levels than all other groups on day 14 (*p* = 0.002) and remained higher than the LYYP-L group on day 28 (*p* = 0.036). Similarly, TGF-α levels in the CON group were significantly higher than in the LYYP-L group on day 14 (*p* = 0.004), and remained higher than in both the LYYP-H and LYYP-L groups on day 28 (*p* = 0.014). Finally, while TGF-β levels on day 14 were significantly elevated in the CON group compared with the other groups (*p* = 0.005), these differences were no longer detectable by day 28 (*p* > 0.05).

### 3.6. Effects of Combined Supplementation of Live Yeast and Yeast Postbiotics on Microbial Community

The alpha diversity indices of the cecal microbiota are summarized in Table 8. Multiple metrics were employed to evaluate community richness (Observed_features and Chao1), diversity (Shannon and Simpson), and evenness (Pielou_e); however, no significant differences were observed among the groups (*p* > 0.05).

**Figure 2:**
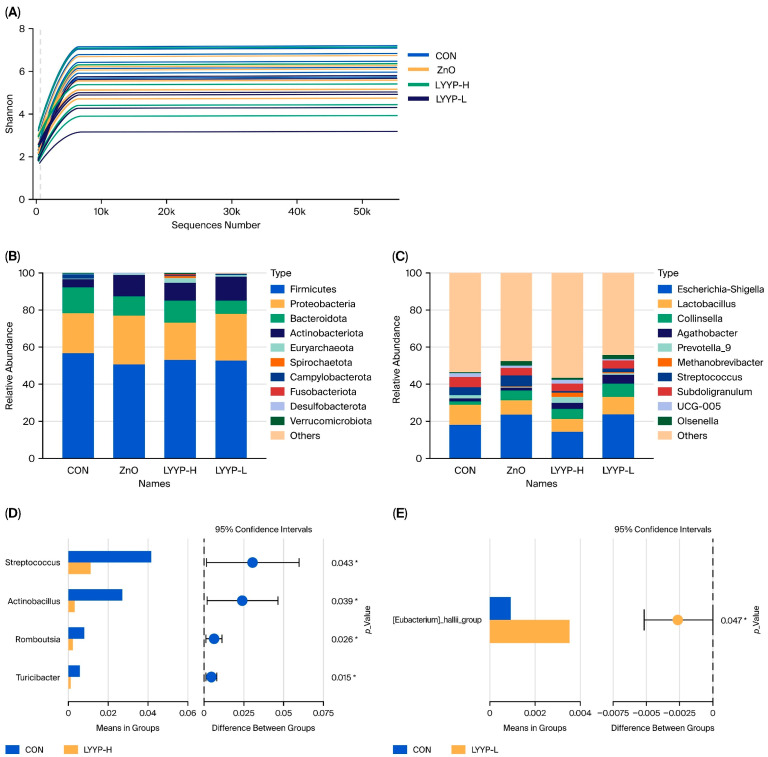
Effects of combined supplementation of live yeast and yeast postbiotics on cecal microbiota composition and diversity. (**A**), rarefaction curve for multi-sample OTU numbers; (**B**), relative abundance on phylum level; (**C**), relative abundance on genus level; (**D**), the species of significant differences at genus level between CON group and LYYP-H group as determined by Wilcoxon rank-sum test; (**E**), the species of significant differences at genus level between CON group and LYYP-L group as determined by Wilcoxon rank-sum test; CON, basal diet; ZnO, basal diet supplemented with 1600 ppm ZnO; LYYP-H, basal diet supplemented with high-dose of live yeast and yeast postbiotics; LYYP-L, basal diet supplemented with low-dose of live yeast and yeast postbiotics. Statistical significance is indicated by * (*p ≤ 0.05*).

As shown in Figure 2, the Shannon diversity rarefaction curve tended to plateau, indicating that the sequencing depth was sufficient to capture the majority of microbial diversity. At the phylum level, *Firmicutes*, *Proteobacteria*, *Bacteroidota*, and *Actinobacteriota* dominated the microbiota across all groups. In the CON group, the mean relative abundance of *Firmicutes* and *Bacteroidota* increased, whereas the relative abundance of *Actinobacteriota* decreased compared to the other groups. At the genus level, notable differences in relative abundance were observed among the groups. In the CON group, the mean relative abundances of *Streptococcus*, *Actinobacillus*, *Romboutsia*, and *Turicibacter* were significantly higher compared with the LYYP-H group, while the relative abundance of *Eubacterium hallii* was significantly lower compared with the LYYP-L group.

## 4. Discussion

Our results indicate that dietary combined supplementation with live yeast and yeast postbiotics did not significantly affect the ADG or ADFI in weaned piglets. The impact of live yeast and yeast postbiotics on growth remains inconsistent across the literature, with various studies yielding conflicting results [14] and others reporting a positive effect of yeast probiotics and postbiotics in poultry [15] and in piglets during the weaning transition [16,17]. In this study, the ADG of the CON group exhibited an upward trend compared with the other groups during Phase II. Notably, the F:G in the LYYP-H group also showed an upward trend during Phase II and was significantly higher than that of the CON and LYYP-L groups throughout the entire trial. Previous research has demonstrated that pharmacological levels of ZnO significantly alter the gut ecosystem by inhibiting *Escherichia coli* overgrowth and promoting the abundance of specific taxa such as *Bacteroidaceae* in weaned piglets [18]. Consequently, the sudden removal of high levels of ZnO likely triggered a secondary shift in the microbial community, forcing the piglets to expend metabolic energy to re-establish intestinal homeostasis. In contrast, piglets in the CON group, which received a consistent basal diet throughout the trial, may have developed a more stable, albeit baseline, microbial equilibrium early on, potentially explaining their steady growth performance in Phase II. On day 28, the LYYP groups exhibited significantly higher antioxidant enzyme activities (SOD and GSH-Px) and sIgA concentrations compared to the CON group. We suggest that this physiological improvement reflects the internal nutrient partitioning priorities of the piglets. As proposed by the ‘immunity cost’ theory [19], maintaining a high-level active defense system requires substantial metabolic expenditure. Consequently, piglets in the LYYP groups, particularly the LYYP-H group, allocated more energy and amino acids toward the protective construction of the active defense system to maintain systemic health, prioritizing immune barrier reinforcement. However, high-dose supplementation of live yeast and yeast postbiotics may have induced slight negative metabolic feedback, resulting in a significantly higher F:G compared to the CON and LYYP-L groups. These findings indicate that during Phase II, under moderate environmental challenges, dietary combined supplementation of high-dose live yeast and yeast postbiotics may not be the most economically viable strategy. In contrast, LYYP-L achieved a balance between maintaining body health and supporting growth performance. Regarding the diarrhea rate, no significant differences were observed among the groups throughout the trial period. Notably, when focusing on the unformed feces rate, dietary combined supplementation with live yeast and yeast postbiotics exhibited better outcomes than the ZnO group and significantly better outcomes than the CON group during both the second phase and the entire trial period, with the LYYP-H group demonstrating the most favorable results. The inclusion of the unformed feces rate as a metric was based on the consideration that a fecal score of 1 may reflect underlying intestinal health issues. This approach aligns with a previous comparative analysis of fecal microbiota between diarrheic and non-diarrheic piglets, which employed the same scoring mechanism and classified individuals with an average fecal score ≥ 1 as the diarrheic group [20]. The first week post-weaning is the most critical period for piglets. However, in this study, fecal consistency was recorded at specific period (Phase I and Phase II), which likely missed the most critical window, especially during Phase II. Therefore, the biological significance of the observed differences in the unformed feces rates might be limited, as the diarrhea rate had already reached a minimal level across all groups.

Oxidative stress is a key factor compromising animal health and growth performance in livestock production [21]. It occurs when free radical production exceeds the capacity of the antioxidant defense system, leading to a redox imbalance [22]. In weanling piglets, multifactorial weaning stressors induce oxidative damage, which subsequently impairs growth performance [23]. As a primary oxidative product of reactive oxygen species (ROS), MDA serves as a key biomarker for assessing the extent of oxidative damage [24]. To counteract oxidative stress, animals utilize an antioxidant defense system consisting of enzymatic and non-enzymatic components. The enzymatic system, primarily including SOD, CAT, and GSH-Px, neutralizes free radicals through sequential biochemical reactions [25]. Consequently, the activities of these enzymes are reliable indicators of body’s antioxidant capacity. Furthermore, nutritional interventions can be employed to enhance this capacity and facilitate the scavenging of free radicals [26]. In this study, dietary combined supplementation of live yeast and yeast postbiotics significantly elevated the activities of SOD and GSH-Px compared with the CON group on both day 14 and day 28. Notably, the activities of SOD and GSH-Px in the high dose of live yeast and yeast postbiotics group also showed an increasing trend compared to the ZnO group, with the GSH-Px activity in the LYYP-H group being significantly higher than that in the ZnO group on day 14. Furthermore, the CAT activity in the ZnO, LYYP-H, and LYYP-L groups exhibited an upward trend compared with the CON group. Additionally, MDA, the end product of lipid peroxidation and a well-established biomarker of high oxidative stress, was significantly higher in the CON group as compared to the levels in the ZnO, LYYP-H, and LYYP-L groups on day 14 and was significantly higher in the CON group as compared to the levels in the LYYP-H group on day 28. Regarding the ZnO group, zinc serves as an essential ion for the catalytic activity of SOD. Consistent with previous findings, zinc supplementation has been shown to enhance intestinal SOD activity in weaned piglets [27]. Furthermore, zinc is intricately involved in the expression of metallothioneins, a class of proteins that exert potent antioxidant effects through their metal-binding capacity [28]. The LYYP groups demonstrated even more pronounced enhancements in antioxidant capacity. Previous studies have indicated that live yeast supplementation can elevate the activities of various antioxidant enzymes [8,29]. Similarly, yeast hydrolysates have been shown to fortify the antioxidant defense system by increasing plasma SOD activity and decreasing MDA concentrations [9]. Our findings further validate that the combination of live yeast and yeast postbiotics exerts a synergistic potentiation of antioxidant capacity. At the molecular level, this synergistic effect may be mediated through the activation of the Keap1-Nrf2-ARE signaling axis. Evidence from previous study in broilers suggests that yeast-derived products markedly upregulate the expression of key antioxidant-related genes, including *Nrf2*, *HO-1*, and *NQO1*, while suppressing their inhibitor, *Keap1* [30]. Notably, the MDA concentration in the LYYP-H group was significantly lower than that of the CON group, which we attribute to the potent radical-scavenging capacity inherent in the combined yeast bioactive components. Evidence from previous research indicates that the supplementation of as little as 0.01% yeast β-glucans in sow diets can significantly mitigate the occurrence of oxidative stress [31], underscoring the low-dose, high-efficiency characteristics of yeast-derived bioactive substances. And the LYYP groups are enriched with β-glucans and yeast-derived peptides, both of which possess potent radical-scavenging capacities [32,33]. The integration of these bioactive constituents provides a robust, multi-component defense mechanism against oxidative challenges. Taken together, these results demonstrate that combined supplementation of live yeast and yeast postbiotics (especially the high-dose supplementation) enhances antioxidant capacity and mitigates oxidative stress in weaned piglets.

The indices of VH, CD, and the V/C ratio serve as primary biological markers for assessing the morphological developmental status and potential digestive capacity of the intestinal mucosa in weaned piglets [34]. Villi are finger-like projections on the intestinal mucosa that expand the surface area available for nutrient absorption [35]. Thus, increased villus height typically correlates with enhanced absorptive efficiency. Crypts, the tubular glands at the base of the villi, reflect cellular proliferation and turnover rates through their depth. The V/C ratio provides a comprehensive assessment of intestinal morphology, where a higher ratio indicates robust intestinal architecture. Previous studies have demonstrated that weaning induces villus atrophy and crypt hyperplasia, thereby compromising the digestive and absorptive functions of piglets [36]. In this study, dietary combined supplementation of live yeast and yeast postbiotics in piglets significantly increased jejunal villus height and the V/C ratio compared with the CON group on both day 14 and day 28, with effects nearly identical to those in the ZnO group. Overall, LYYP-L group exhibited superior improvements in intestinal morphology compared with LYYP-H group. These results indicate that the combination of live yeast and yeast postbiotics can reverse the deleterious effects of weaning on intestinal morphology, ensuring the healthy development of the gastrointestinal tract in piglets.

The expression of tight-junction genes, such as *ZO-1*, *Claudin-1*, and *Occludin*, is essential for excluding pathogens and maintaining intestinal homeostasis [37,38]. However, no significant differences in the expression levels of these three genes were observed among the groups. While DAO is an intracellular enzyme that facilitates mucosal repair, D-LA is a bacterial metabolite that accumulates during dysbiosis and bacterial overgrowth [39,40]. While plasma concentration of D-LA and DAO activity are conventional markers of intestinal barrier damage, this study quantified these parameters specifically within the intestinal mucosa. A previous study investigating the effects of cysteamine supplementation on porcine ileal mucosal health similarly measured ileal mucosal DAO activity, utilizing it as an indicator of intestinal morphology and homeostasis [41]. Furthermore, we believe that monitoring local mucosal D-LA levels facilitates the assessment of the bacterial metabolic load within the microenvironment of the mucosal surface. Additionally, no significant differences in DAO or D-LA levels were observed among the groups. In summary, dietary combined supplementation with live yeast and yeast postbiotics did not significantly influence overall intestinal barrier integrity, which was consistent with the observations in the ZnO group.

Intestinal immunological markers are equally vital for assessing intestinal health [42]. The antibody sIgA plays a fundamental role in mucosal defense by binding to pathogens and commensal bacteria, thereby preventing their direct contact with the intestinal epithelium. This sequestering mechanism is essential for disease prevention and the maintenance of immune homeostasis [43]. In this study, dietary supplementation with live yeast and yeast postbiotics significantly increased the sIgA content in piglets compared with the CON group on day 28, with effects nearly identical to those observed in the ZnO group. Furthermore, sIgA level in the CON group decreased significantly in the second phase compared to the first. These findings suggest a potential bacterial infection within the CON group during the latter phase, where sIgA was likely depleted through binding with bacterial pathogens. As a Th1-type pro-inflammatory cytokine, IFN-γ typically elevates in cases of intestinal inflammation disease. Similar to IFN-γ, TNF-α is closely linked to the pathogenesis of intestinal inflammation. Both IFN-γ and TNF-α directly compromise tight-junction integrity, thereby increasing intestinal permeability [44]. In this study, the IFN-γ and TNF-α contents in the CON group were significantly higher than that in the other groups on both day 14 and day 28, possibly indicating that the intestinal tract of piglets in the CON group faced greater bacterial or viral challenges. This led to an active intestinal immune system, resulting in intestinal inflammatory responses. Notably, the levels of IFN-γ and TNF-α in the LYYP groups exhibited a downward trend compared to the ZnO group on day 28, suggesting that dietary supplementation with live yeast and yeast postbiotics may possess a superior capacity for modulating inflammatory responses. Additionally, the pro-inflammatory cytokine IL-6 is secreted in large quantities upon exposure to pro-inflammatory stimuli [45]. In this study, the level of IL-6 in the CON group was also significantly elevated, corresponding to the increase in IFN-γ and TNF-α. These findings suggest that immune cells of piglets in the CON group were highly active, exhibiting a pro-inflammatory predisposition, while piglets in LYYP groups have a healthy gut with more stable immune response. Previous research has also demonstrated that yeast and its derivatives possess the capacity to suppress the expression of pro-inflammatory cytokines and alleviate inflammatory responses [11]. The anti-inflammatory cytokine IL-10 plays a pivotal role in modulating excessive immune responses. By inhibiting the synthesis and release of pro-inflammatory mediators, IL-10 prevents tissue damage and preserves the structural integrity of the intestinal epithelium [46]. In the CON group, the IL-10 level was significantly elevated alongside those pro-inflammatory cytokines, likely serving as a homeostatic regulation mechanism to counterbalance the high levels of pro-inflammatory cytokines and prevent severe intestinal damage. In terms of mucosal recovery, TGF-α acts as a growth factor that promotes epithelial cell proliferation and repair [47]. As a pleiotropic cytokine, TGF-β exerts potent immunosuppressive effects while simultaneously promoting connective tissue proliferation and collagen synthesis, thereby facilitating tissue repair and structural recovery [48]. In this study, the abundant expression of TGF-α and TGF-β in the intestinal mucosa of the CON group likely reflects a physiological effort to promote intestinal repair and mucosal remodeling in response to weaning-induced challenges. This interpretation is consistent with the morphological findings, as the VH in the CON group remained significantly lower than that of the other groups on both day 14 and day 28. Consistent with the superior inflammatory control observed in the LYYP groups, the levels of TGF-α and TGF-β also exhibited a downward trend compared to the ZnO group on day 14. Previous study has also reported that immunoglobulin levels are enhanced by *Saccharomyces cerevisiae* [49].

The experimental findings indicate that dietary supplementation with live yeast and yeast postbiotics did not significantly affect the gut microbiota diversity in piglets. However, the abundances of *Streptococcus*, *Actinobacillus*, *Romboutsia*, and *Turicibacter* were significantly reduced in the LYYP-H group compared with the CON group, with some species within these genera identified as potential pathogens. For instance, *Actinobacillus* is a well-known opportunistic pathogen; some species within this genus are responsible for several distinct pathological conditions in animals [50]. Similarly, *Streptococcus suis* is a zoonotic pathogen capable of infecting both swine and humans, frequently manifesting as septicemia and meningitis [51]. The reduction in these opportunistic pathogens is likely driven by the abundance of MOS in live yeast and yeast postbiotics. The structural similarity between MOS and the bacterial attachment sites on intestinal epithelial cells allows MOS to competitively inhibit the adherence and colonization of pathogens. In contrast, the abundance of the *Eubacterium hallii* was significantly increased in the LYYP-L group compared with the CON group. This microbial genus is recognized for its ability to produce butyrate, a short-chain fatty acid with anti-inflammatory properties. Butyrate plays a critical role in regulating intestinal immune responses and suppressing the release of inflammatory cytokines [52]. These findings underscore the need for further investigation into the underlying mechanisms to better understand and elucidate the specific effects of live yeast and yeast postbiotics on the intestinal health of piglets.

Despite these meaningful findings, several limitations of the present study warrant consideration. First, the relatively small sample size (n = 7 replicates per group) represents a primary constraint, which limits statistical power and increases the potential risk of Type I and Type II errors in both growth performance and physiological analyses. Second, the abrupt withdrawal of pharmacological ZnO in Phase II—while mandated by regulatory compliance—introduced a discrepancy in treatment continuity. This lack of uniformity in supplementation duration serves as a potential confounding factor, complicating the direct comparison of long-term efficacy between the ZnO and LYYP groups. Third, the independent analysis of time points, rather than utilizing a mixed-effects model for repeated measures, may have overlooked crucial diet-time interactions. Finally, our sampling schedule (day 14 and day 28) likely missed the acute phase of weaning stress, which typically peaks within the first week post-weaning. Furthermore, the absence of intestinal oxidative markers and nutrient digestibility data limit a more comprehensive evaluation of the protective effects of live yeast and yeast postbiotics. Future studies incorporating earlier sampling windows and broader physiological parameters are required to validate these outcomes.

## 5. Conclusions

This study has clearly shown that dietary combined supplementation with live yeast and yeast postbiotics significantly enhanced fecal consistency in weaned piglets to a level comparable to that of 1600 ppm ZnO. Moreover, the LYYP groups exhibited enhanced antioxidant and immunological capacities, as well as improved intestinal morphology. Additionally, the combination of high-dose live yeast and yeast postbiotics may reduce the abundance of several potentially harmful microorganisms in the cecum of piglets, while low-dose supplementation may increase the abundance of beneficial microorganisms in the cecum. Although growth performance remained unchanged during the trial phase, the observed enhancements in antioxidant capacity and intestinal health suggest that combination of live yeast and yeast postbiotics presents a viable alternative to the 1600 ppm ZnO supplementation during the first two weeks post-weaning, as growth performance in the LYYP groups was not negatively affected during this initial period.

## Figures and Tables

**Table 1:** Ingredient composition and nutritional values of the basal diets.

Items	Basal Diet I	Basal Diet II
Ingredients (%)		
Corn	42.055	47.005
Extruded corn	15.000	15.000
Rice bran meal	4.000	4.000
Sucrose	2.000	2.000
Extruded soybean	0.000	3.800
Soymilk powder	5.000	0.000
Soy oil	2.500	1.000
Dehulled soybean meal	11.900	16.500
Fish meal	2.500	2.500
High-protein whey powder	10.000	5.000
Dicalcium phosphate	0.500	0.500
Limestone	0.550	0.800
NaCl	0.300	0.400
L-Lysine HCL	0.500	0.460
DL-Methionine	0.150	0.100
L-Threonine	0.200	0.150
L-Tryptophan	0.060	0.050
Phytase	0.025	0.025
Tributyrin	0.200	0.200
Yeast protein	2.000	0.000
Hemicell	0.010	0.010
Vitamin-mineral premix ^a^	0.500	0.500
Calculated Values (%, DM)		
Metabolizable energy (MJ/kg)	14.64	14.25
Crude protein	18.53	17.34
Crude fat	4.60	4.00
Calcium	0.63	0.71
Total Zn (mg/kg)	101.70	105.78
Total phosphorus	0.56	0.55
SID ^b^ Lys	1.29	1.15
SID Met	0.42	0.36

^a^ Vitamin and mineral premix provided the following per kilogram of diet: 12,000 IU vitamin A as vitamin A acetate, 2500 IU vitamin D as vitamin D3, 30 IU vitamin E as dl-α-tocopheryl acetate, 12 μg vitamin B12, 3 mg vitamin K as menadione sodium bisulfate, 15 mg d-pantothenic acid as calcium pantothenate, 40 mg nicotinic acid as nicotinamide, 400 mg choline as choline chloride, 30 mg Mn as manganese oxide, 90 mg Fe as iron sulfate, 80 mg Zn as zinc oxide, 10 mg Cu as copper sulfate, 0.35 mg I as ethylenediamine dihydroiodide, and 0.3 mg Se as sodium selenite. ^b^ Standardized ileal digestible.

**Table 2:** Primers used for quantitative real-time PCR to determine the expression of intestinal tissue tight junction-related genes.

Gene	Primer Sequence (5′ → 3′)	Product Length	Gene Accession Number
*β-actin*	F: TACGCCAACACGGTGCTGTC	207 bp	NM_001444420.1
	R: GTACTCCTGCTTGCTGATCCACAT		
*ZO-1*	F: GAGGATGGTCACACCGTGGT	169 bp	NM_001444400.1
	R: GGAGGATGCTGTTGTCTCGG		
*Occludin*	F: ATGCTTTCTCAGCCAGCGTA	176 bp	NM_001244539.1
	R: AAGGTTCCATAGCCTCGGTC		
*Claudin-1*	F: CAAAACCTTCGCCTTCCAG	293 bp	NM_001163647.2
	R: TCCCCACATTCGAGATGATTAC		

Abbreviations: *ZO-1*, zonula occludens-1.

**Table 3:** Effects of combined supplementation of live yeast and yeast postbiotics on growth performance and diarrhea incidence in weaned piglets.

Items	CON	ZnO	LYYP-H	LYYP-L	SEM	*p*
Phase I						
ADG (g/d)	328.13	348.43	325.65	329.94	5.88	0.359
ADFI (g/d)	472.66	488.59	475.51	479.28	12.18	0.974
F:G	1.44	1.42	1.51	1.48	0.02	0.427
diarrhea rate (%)	1.23	0.74	0.50	0.97	/	0.412
unformed feces rate (%)	5.54	4.68	3.11	3.87	/	0.090
Phase II						
ADG (g/d)	407.79	360.29	354.76	383.89	8.21	0.085
ADFI (g/d)	736.38	689.14	706.12	698.90	18.39	0.837
F:G	1.80	1.85	2.00	1.80	0.03	0.077
diarrhea rate (%)	0.86	1.28	0.30	0.56	/	0.168
unformed feces rate (%)	4.71 ^a^	3.26 ^ab^	2.08 ^b^	2.38 ^b^	/	0.026
Entire trial period						
ADG (g/d)	373.54	357.14	338.91	355.67	6.38	0.304
ADFI (g/d)	604.52	588.87	590.81	589.09	14.69	0.981
F:G	1.61 ^b^	1.66 ^ab^	1.74 ^a^	1.64 ^b^	0.02	0.005
diarrhea rate (%)	1.10	0.99	0.41	0.78	/	0.152
unformed feces rate (%)	5.16 ^a^	4.02 ^ab^	2.64 ^b^	3.18 ^b^	/	0.002

Abbreviations: CON, basal diet; ZnO, basal diet supplemented with 1600 ppm ZnO; LYYP-H, basal diet supplemented with high-dose of live yeast and yeast postbiotics; LYYP-L, basal diet supplemented with low-dose of live yeast and yeast postbiotics; ADG, average daily gain; ADFI, average daily feed intake; F:G, the ratio of feed intake to gain (higher F:G indicates worse feed efficiency). ^a,b^ Means within a row with different letters differed significantly (*p* < 0.05) as determined by Tukey’s test. / Means SEM is not provided for diarrhea incidence as they were calculated and analyzed as categorical frequency data.

**Table 4:** Effects of combined supplementation of live yeast and yeast postbiotics on serum antioxidant indices in weaned piglets.

Items	CON	ZnO	LYYP-H	LYYP-L	SEM	*p*
14 d						
SOD (U/mL)	130.50 ^b^	151.62 ^a^	146.70 ^a^	143.85 ^ab^	1.91	0.001
CAT (U/mL)	6.76	8.69	9.00	8.38	0.34	0.089
GSH-Px (U/mL)	182.78 ^b^	183.39 ^b^	227.01 ^a^	204.91 ^ab^	5.02	0.001
MDA (nmol/mL)	4.84 ^a^	3.40 ^b^	3.75 ^b^	3.82 ^b^	0.12	0.001
28 d						
SOD (U/mL)	138.61 ^b^	153.41 ^a^	163.16 ^a^	150.59 ^ab^	0.48	0.025
CAT (U/mL)	7.65	9.83	9.76	9.60	0.36	0.089
GSH-Px (U/mL)	185.03 ^b^	250.52 ^a^	270.91 ^a^	243.28 ^a^	7.63	0.001
MDA (nmol/mL)	3.75 ^a^	2.91 ^ab^	2.38 ^b^	3.09 ^ab^	0.14	0.003

Abbreviations: CON, basal diet; ZnO, basal diet supplemented with 1600 ppm ZnO; LYYP-H, basal diet supplemented with high-dose of live yeast and yeast postbiotics; LYYP-L, basal diet supplemented with low-dose of live yeast and yeast postbiotics; SOD, superoxide dismutase; CAT, catalase; GSH-Px, glutathione peroxidase; MDA, malondialdehyde. ^a,b^ Means within a row with different letters differed significantly (*p* < 0.05) as determined by Tukey’s test.

**Table 5:** Effects of combined supplementation of live yeast and yeast postbiotics on intestinal morphology in weaned piglets.

Items	CON	ZnO	LYYP-H	LYYP-L	SEM	*p*
14 d						
VH (μm)	361.97 ^b^	590.99 ^a^	603.07 ^a^	634.04 ^a^	32.07	0.002
CD (μm)	475.88	499.79	430.86	442.84	18.65	0.573
V/C	0.80 ^b^	1.23 ^ab^	1.33 ^a^	1.50 ^a^	0.08	0.011
28 d						
VH (μm)	421.16 ^b^	714.66 ^a^	556.48 ^ab^	628.03 ^a^	32.46	0.004
CD (μm)	516.49	497.91	470.89	414.66	19.13	0.255
V/C	0.86 ^b^	1.44 ^a^	1.24 ^ab^	1.52 ^a^	0.08	0.004

Abbreviations: CON, basal diet; ZnO, basal diet supplemented with 1600 ppm ZnO; LYYP-H, basal diet supplemented with high-dose of live yeast and yeast postbiotics; LYYP-L, basal diet supplemented with low-dose of live yeast and yeast postbiotics; VH, villus height; CD, crypt depth; V/C, villus height:crypt depth. ^a,b^ Means within a row with different letters differed significantly (*p* < 0.05) as determined by Tukey’s test.

**Table 6:** Effects of combined supplementation of live yeast and yeast postbiotics on DAO and D-LA levels in weaned piglets.

Items	CON	ZnO	LYYP-H	LYYP-L	SEM	*p*
14 d						
DAO (U/mg)	8.30	8.30	7.30	7.00	0.480	0.683
D-LA (µmol/mg)	0.79	0.71	0.74	0.69	0.025	0.601
28 d						
DAO (U/mg)	5.20	5.10	4.00	4.80	0.260	0.365
D-LA (µmol/mg)	0.69	0.71	0.74	0.81	0.029	0.371

Abbreviations: CON, basal diet; ZnO, basal diet supplemented with 1600 ppm ZnO; LYYP-H, basal diet supplemented with high-dose of live yeast and yeast postbiotics; LYYP-L, basal diet supplemented with low-dose of live yeast and yeast postbiotics; DAO, diamine oxidase; D-LA, D-lactate.

**Table 7:** Effects of combined supplementation of live yeast and yeast postbiotics on immune indices in weaned piglets.

Items	CON	ZnO	LYYP-H	LYYP-L	SEM	*p*
14 d						
sIgA (ng/g)	0.45	0.46	0.43	0.40	0.02	0.717
IFN-γ (ng/g)	22.40 ^a^	15.10 ^b^	17.26 ^ab^	17.58 ^ab^	0.86	0.017
TNF-α (pg/g)	20.60 ^a^	13.20 ^b^	12.20 ^b^	13.30 ^ab^	0.87	0.004
IL-6 (ng/g)	74.80 ^a^	42.40 ^b^	53.50 ^ab^	51.10 ^ab^	3.50	0.012
IL-10 (ng/g)	28.30 ^a^	16.50 ^b^	16.40 ^b^	16.60 ^b^	1.10	0.002
TGF-α (pg/g)	12.75 ^a^	8.48 ^ab^	7.92 ^ab^	6.79 ^b^	0.54	0.004
TGF-β (ng/g)	172.30 ^a^	110.40 ^ab^	93.70 ^b^	85.50 ^b^	8.47	0.005
28 d						
sIgA (ng/g)	0.30 ^b^	0.44 ^a^	0.45 ^a^	0.43 ^a^	0.02	0.003
IFN-γ (ng/g)	21.03 ^a^	18.02 ^ab^	14.15 ^b^	14.29 ^b^	0.84	0.004
TNF-α (pg/g)	14.22 ^a^	11.56 ^ab^	11.05 ^b^	10.08 ^b^	0.44	0.002
IL-6 (ng/g)	52.33 ^a^	39.33 ^b^	41.07 ^b^	45.01 ^ab^	1.63	0.021
IL-10 (ng/g)	19.08 ^a^	15.63 ^ab^	16.52 ^ab^	14.50 ^b^	0.60	0.036
TGF-α (pg/g)	9.39 ^a^	7.26 ^ab^	5.92 ^b^	5.53 ^b^	0.48	0.014
TGF-β (ng/g)	93.25	88.59	72.43	82.29	3.46	0.170

Abbreviations: CON, basal diet; ZnO, basal diet supplemented with 1600 ppm ZnO; LYYP-H, basal diet supplemented with high-dose of live yeast and yeast postbiotics; LYYP-L, basal diet supplemented with low-dose of live yeast and yeast postbiotics; sIgA, secretory immunoglobulin A; IFN-γ, interferon-γ; TNF-α, tumor necrosis factor-α; IL-6, interleukin-6; IL-10, interleukin-10; TGF-α, transforming growth factor-α; TGF-β, transforming growth factor-β. ^a,b^ Means within a row with different letters differed significantly (*p* < 0.05) as determined by Tukey’s test.

**Table 8:** Effects of combined supplementation of live yeast and yeast postbiotics on alpha diversity indices in weaned piglets.

Items	CON	ZnO	LYYP-H	LYYP-L	SEM	*p*
28 d						
Shannon	5.96	5.45	5.99	5.47	0.18	0.430
Chao1	467.96	434.72	484.87	445.30	21.09	0.741
Simpson	0.94	0.93	0.93	0.93	0.01	0.906
Observed_features	464.14	428.57	478.86	441.57	20.92	0.743
Pielou_e	0.68	0.63	0.67	0.67	0.02	0.503

Abbreviations: CON, basal diet; ZnO, basal diet supplemented with 1600 ppm ZnO; LYYP-H, basal diet supplemented with high-dose of live yeast and yeast postbiotics; LYYP-L, basal diet supplemented with low-dose of live yeast and yeast postbiotics. Alpha diversity indices were assessed only at a single time point (day 28).

## Data Availability

Data are available from the corresponding author on reasonable request.

## Supplementary Materials

The following supporting information can be downloaded at: https://www.mdpi.com/article/10.3390/antiox15050623/s1, Figure S1. Representative qPCR amplification and melting curves of target genes on day 14. Figure S2. Representative qPCR amplification and melting curves of target genes on day 28.

## Abbreviations

The following abbreviations are used in this manuscript:

ADFI	Average daily feed intake
ADG	Average daily gain
CAT	Catalase
CD	Crypt depth
F:G	Feed intake to gain ratio
GSH-Px	Glutathione peroxidase
IL-10	Interleukin-10
IL-6	Interleukin-6
IFN-γ	Interferon-γ
MDA	Malondialdehyde
MOS	Mannan-oligosaccharides
OTUs	Operational taxonomic units
PCoA	Principal coordinate analysis
ROS	Reactive oxygen species
sIgA	Secretory immunoglobulin A
SID Lys	Standardized ileal digestible lysine
SID Met	Standardized ileal digestible methionine
SOD	Superoxide dismutase
TGF-α	Transforming growth factor-α
TGF-β	Transforming growth factor-β
TNF-α	Tumor necrosis factor-α
V/C	Villus height:crypt depth
VH	Villus height
ZnO	Zinc oxide
ZO-1	Zonula occludens-1
