# Supplementary material for: Combined Supplementation of Live Yeast and Yeast Postbiotics Enhances Antioxidant Capacity and Intestinal Health in Weaned Piglets
Source: Antioxidants (Basel). 2026 May 14;15(5):623. doi: 10.3390/antiox15050623 (PMC13203434; doi:10.3390/antiox15050623)
Supplement: Supplementary file 1 [file antioxidants-15-00623-s001.zip › antioxidants-4241069-supplementary.pdf]

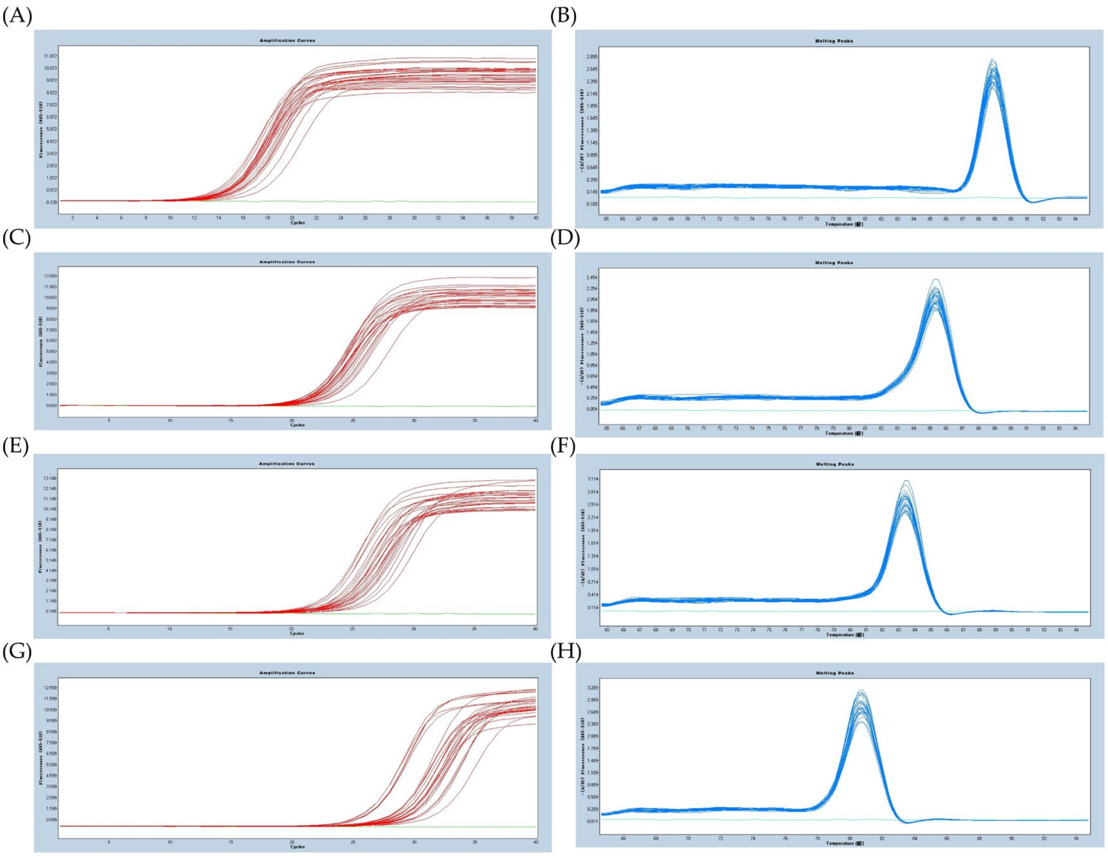

Figure S1. Representative qPCR amplification and melting curves of target genes on day 14. (A) Amplification curve and (B) melting curve of *β-actin*; (C) Amplification curve and (D) melting curve of zonula occludens-1; (E) Amplification curve and (F) melting curve of *Occludin*; (G) Amplification curve and (H) melting curve of *Claudin-1*. The amplification curves (A, C, E, G) exhibit high technical reproducibility and consistent slopes during the exponential phase. The melting curves (B, D, F, H) show single, sharp dissociation peaks at specific temperatures, confirming the high specificity of the primers.

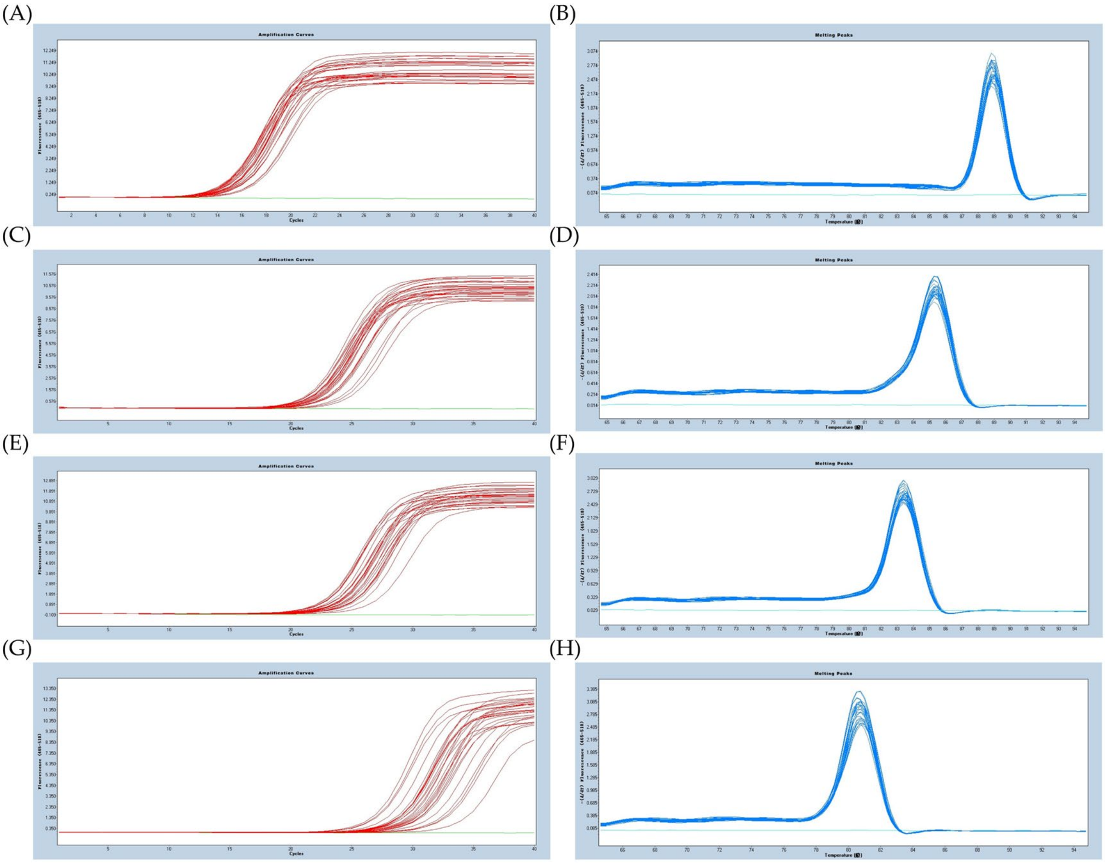

Figure S2. Representative qPCR amplification and melting curves of target genes on day 28. (A) Amplification curve and (B) melting curve of *β-actin*; (C) Amplification curve and (D) melting curve of zonula occludens-1; (E) Amplification curve and (F) melting curve of *Occludin*; (G) Amplification curve and (H) melting curve of *Claudin-1*. The amplification curves (A, C, E, G) exhibit high technical reproducibility and consistent slopes during the exponential phase. The melting curves (B, D, F, H) show single, sharp dissociation peaks at specific temperatures, confirming the high specificity of the primers.
